# Estimation of Total Daily Fluoride Intake in Europe and Correlation to Equivalent Doses in Epidemiological Studies

**DOI:** 10.1002/jat.4865

**Published:** 2025-07-22

**Authors:** Stefanie Scheffler, Falko Partosch, Ariane Zwintscher, Annette Bitsch

**Affiliations:** ^1^ Fraunhofer ITEM Hannover Germany

**Keywords:** chronic fluoride exposure, dose estimation, equivalent intake, exposure assessment, fluoride toxicity

## Abstract

European populations are chronically exposed to fluoride, as fluoride is supplemented for caries prophylaxis and is furthermore present in some food sources. As there is evidence that fluoride exposure at drinking water concentrations above 1.5 mg/L is associated with lower IQ in children, total daily intake in Europe might be close to or above this exposure level. Concerning health effects in Europe, epidemiological data are limited. Therefore, it would be beneficial to consider existing studies from non‐EU countries to transfer observed effects to the exposure situation in Europe. Additionally, animal data could also deliver supporting information, if equivalent doses could be calculated. In this work, a methodology was developed to determine daily fluoride intake and excretion in Europe and align it to concentrations reported in animal and epidemiological studies. With this, a total daily intake of 2.05‐mg fluoride for 3‐year‐old children and 3.8‐mg fluoride for adults was estimated. For 3‐year‐olds, this value exceeds the current recommendations of fluoride intake by EFSA and even the tolerable upper intake level. The daily urinary fluoride excretion was calculated to be 0.72 mg/day, and for adults, it was 2.05 mg/day. For in vivo studies, rat exposure to fluoride drinking water concentrations of 12.5 ppm was considered to be equivalent to the daily fluoride intake in Europe. With the presented approach, equivalent doses can be applied to select international epidemiological as well as in vivo studies reflecting the fluoride exposure situation in European countries to extrapolate potential health effects.

## Abstract 2

European populations are chronically exposed to fluoride through dental prophylaxis and diet. Evidence links fluoride exposure at drinking water concentrations above 1.5 mg/L to reduced IQ in children. This study developed a method to estimate daily fluoride intake and excretion in Europe, finding levels that exceed EFSA recommendations for children. Equivalent doses were derived to align with animal and international epidemiological studies. This approach enables extrapolation of potential health effects from international epidemiological and animal studies to the European context.

## 1 Introduction

Long‐term exposure to high fluoride doses has long been known to induce severe health issues as observed in regions having naturally high groundwater concentrations such as India, China, and Mexico. In those regions, being located in one of the world's five fluoride belts,^1^ where fluoride drinking water concentrations can be above 6 mg/L (Chowdhury et al. 2019), severe dental and crippling fluorosis can be observed, and fertility issues have been reported as well (Choubisa 2012; NRC 1993). Several epidemiological studies are dealing with health issues associated with chronic exposure to high fluoride concentrations in these areas (Chahal et al. 2014; Chauhan et al. 2013; Choubisa 2012; Duan et al. 2016; Godebo et al. 2020; Jaganmohan et al. 2010; Koroglu et al. 2011; Liu et al. 2014, 2019; Ortiz‐Pérez et al. 2003; Quadri et al. 2018; Rango et al. 2014, 2017; Shashi and Singla 2013; Sun et al. 2013; Wasana et al. 2016; Wu et al. 2019; Yasmin et al. 2014; Zhao et al. 2015; Zhou et al. 2012).

In 2006, the National Research Council (NRC) first published an evaluation suggesting an association between high levels of fluoride in drinking water and adverse neurological effects in humans (NRC 2006). Since then, many studies have been published dealing with this topic. Recently, assessments by the National Toxicology Program (NTP) and Health Canada conclude with moderate confidence that fluoride exposure at drinking water concentrations above 1.5 mg/L is associated with lower IQ in children (NTP 2024; Health Canada 2023).

In European countries, the current legal limit for drinking water in Europe is 1.5 mg/L and therefore close to the threshold suggested by NRC, although actual drinking water levels in Europe are generally below 0.3‐mg/L fluoride. However, total fluoride exposure is higher than through drinking water alone. European populations are chronically exposed to fluoride since oral exposure occurs voluntarily or by regulation through fluoridated salt (Götzfried 2006) as well as further sources such as fluoridated toothpaste. Furthermore, fluoride is inherently present in some food sources, such as fish or black tea, increasing intake further. Therefore, the total daily intake in Europe might be close to or above the intake through drinking water, with 1.5 mg/L fluoride being associated with adverse health effects.

However, epidemiological studies regarding adverse health effects through chronic fluoride exposure in Europe are sparse. In the NTP report, only two studies were cited from Europe. Therefore, more data are needed to address possible adverse health effects for this distinct exposure situation.

Since numerous epidemiological studies are already available from non‐EU countries, dealing with health effects through fluoride exposure, their use for the European context would be advantageous. The majority of these studies have been performed in regions with high levels of naturally occurring fluoride in drinking water or community water fluoridation outside of Europe. Here, the exposure situation is different, as the main fluoride source is fluoridated water and not salt, as it is the situation in Europe. Therefore, single intake dosages as well as intake frequency differ. To relate effects observed in existing studies to the specific situation in Europe, the total daily fluoride intake needs to be comparable. By using a marker of total intake, such as urinary fluoride excretion, intake can be compared regardless of the different sources.

In addition to epidemiological studies, many in vivo studies on the influence of fluoride on animal health have been published, which may provide valuable supportive insights. To make these data comparable, a correlation of the intake across the different species is required. Since animals are typically exposed to fluoride via drinking water, a direct comparison can be made to human epidemiological studies with the same exposure route. The corresponding drinking water level in humans can further be compared to the European situation by using urinary fluoride excretion as a marker of fluoride intake, in order to finally determine equivalent fluoride exposure levels in rats.

To address all mentioned aspects, the following approach was taken in this study:

1. Determination of daily fluoride intake in Europe
2. Determination of daily urinary excretion in Europe to correlate the intake situation to epidemiological studies
3. Correlation of fluoride intake from rat studies to epidemiological studies with drinking water exposure and further alignment to fluoride intake in Europe

## 2 Methods

### 2.1 Determination of Realistic Daily Fluoride Intake

**FIGURE 1:**
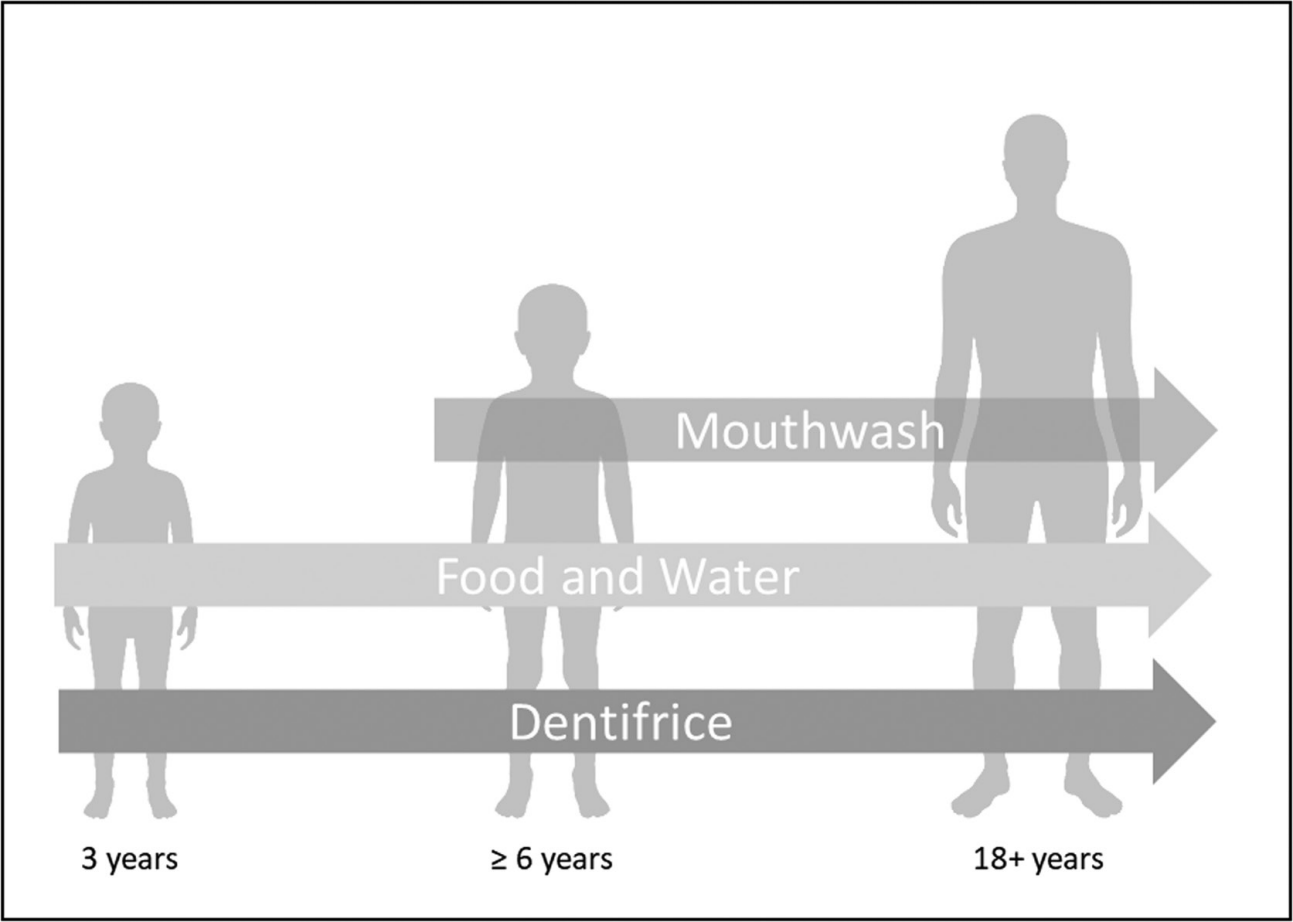
Main sources of fluoride supplementation for different age groups.

In countries with low natural fluoride occurrence, fluoride is supplemented for caries prophylaxis in different ways. Besides toothpaste, fluoridated salt is the main source, but fluoride‐containing mouthwash can also contribute. Although drinking water contains only low natural fluoride levels, it also adds to overall intake as well as food with high fluoride content, such as fish and black tea. Fluoride tablets are only recommended in specific situations. For different age groups, the share of the single sources differs substantially as presented schematically in Figure 1.

For 3‐year‐old children, drinking water and toothpaste (used twice a day as recommended) are assumed to be the main fluoride sources, but fish consumption can contribute to the total intake as well. Mouthwash is not recommended, intake through salt is low in this age group, and black tea is not considered relevant. Fluoride tablets are only recommended if no fluoride toothpaste is used (BfR 2018; DAKJ 2007).

With increasing age, other fluoride sources gain more importance as, dependent on water fluoride concentrations, the usage of fluoridated salt or fluoride tablets is recommended (in Germany) to reach a certain daily fluoride dose (DAKJ 2007).

For children 6 years and older as well as adults, it is assumed that exposure to fluoride takes place through toothpaste, drinking water, and food (due to fish, tea and fluoridated salt).

Usage of fluoride tablets is not common in children above 4 years and adults since they are only recommended as an alternative to fluoridated salt (BgVV 2002). Therefore, they are not additionally considered for the calculation of the daily worst‐case intake.

Furthermore, for children above 6 years and adults, mouth wash can contribute to the total intake. For adults, black tea consumption can further increase the total intake.

Therefore, the daily total fluoride intake depends on the age group and within the group on the intake of different fluoride sources and amounts (e.g., drinking water, food, supplements, toothpaste/oral care products). To calculate a realistic range of daily intake levels in Europe, the recommendations for toothpaste usage as published by the European Academy of Paediatric Dentistry and, additionally, studies about toothpaste dosage and ingestion served as a first approach to assess an exposure range (Barnhart et al. 1974; Cochran et al. 2004; Creeth et al. 2013; de Almeida et al. 2007; Franco et al. 2005; Martínez‐Mier et al. 2003; Naccache et al. 1992; Omid et al. 2017; Rojas‐Sanchez et al. 1999; Stoye‐Herzog et al. 2009; Thornton‐Evans et al. 2019; Toumba et al. 2019; van Loveren et al. 2004; Wiener et al. 2009; Zohoori et al. 2012). Moreover, exposure through other sources such as food, drinking water, and mouthwash was additionally regarded. Foods other than those supplemented with fluoride through salt were not considered as they contain only very small amounts of fluoride. An exception to this is fish and seafood as well as black tea, which can contain high amounts of fluoride and are therefore considered.

### 2.2 Determination of Equivalent Exposure Ranges in Epidemiological Studies

Most epidemiological studies evaluating fluoride exposure were performed in regions with naturally high fluoride concentrations in groundwater or community water fluoridation outside of Europe. In contrast to Europe, where salt is the major fluoride source, in these regions, the fluoride is mainly delivered through drinking water, and fluoride doses as well as exposure frequency differ substantially.

In order to correlate epidemiological studies to the exposure situation in Europe, the total daily fluoride intake must be aligned.

As in some epidemiological studies, sources other than water might not be included or quantitatively determined, the daily urinary fluoride concentration is a more accurate indicator of actual total fluoride intake and is therefore considered for correlation purposes.

### 2.3 Determination of Equivalent Doses in Animal Studies

To compare fluoride doses applied to rats in in vivo studies with the fluoride exposure situation in humans in Europe, a reverse estimation of exposure has been performed based on plasma levels of rats and humans with dental fluorosis.

As animals are mostly exposed to fluoride through drinking water, first, a correlation was made to epidemiological studies with drinking water exposure. Subsequently, the determined dose from epidemiological studies was correlated to the European situation to finally determine an equivalent dose in animal studies.

## 3 Results and Discussion

### 3.1 Determination of Realistic Daily Fluoride Intake

#### 3.1.1 Detailed Data on Fluoride Ingestion via Toothpaste Usage

According to the European Academy of Paediatric Dentistry, different amounts of toothpaste are recommended for children depending on their age (Toumba et al. 2019). For children up to 2 years, a smear (or size of a grain of rice, 0.125 g) should be applied, and for children 2–6 years, a pea‐sized amount (0.25 g) is recommended. For children up to 6 years, a 1000‐ppm toothpaste is recommended to be used twice a day. For over 6‐year‐olds and adults, up to a full load (0.5–1 g) of a toothpaste containing 1450 ppm should be used twice a day.

However, dispensing the right dosage is challenging for children and caregivers. Several researchers studied whether recommendations can actually be implemented by children and parents and found that toothpaste is often overdosed. For children up to 6 years, applications of 0.5 g, 1 g, or even more are common (Creeth et al. 2013; Martínez‐Mier et al. 2003; Stoye‐Herzog et al. 2009; Thornton‐Evans et al. 2019; van Loveren et al. 2004; Wiener et al. 2009).

Besides amounts being higher than recommended, it was found that children have high ingestion rates. Researchers analyzed expectorated toothpaste and residues left on the toothbrush and found that the ingestion decreases with age due to improving disgorging skills. Consequently, young children have the highest rates of ingestion. For 1‐ to 3.5‐year‐old children, rates were reported to be 43%–85.7% (Cochran et al. 2004; de Almeida et al. 2007; Franco et al. 2005; Naccache et al. 1992; Rojas‐Sanchez et al. 1999; van Loveren et al. 2004).

Ingestion rates of older children and adults are less frequently studied, but for 6‐year‐old children, rates of 13.9%–36% have been given (Barnhart et al. 1974; Zohoori et al. 2012). One study reported a mean ingestion rate of 2.9% for adults 20–35 years of age (Barnhart et al. 1974). This value is in accordance with newer data, reporting that ingestion in adults is less than 10% (European Commission 2010).

Based on the given data, a consumption of 1 g toothpaste twice a day is considered for all age groups. Furthermore, worst case ingestion rates are considered, being 80% for children up to 3 years, 36% for 6‐year‐old children, 30% for 8‐ to 10‐year‐old children (extrapolated value based on data for 6‐year‐olds, as no data were available for this age group), and 3% for adults (data given in Table 1).

**TABLE 1:** Data for toothpaste use considered for the calculation of the daily fluoride intake to represent a realistic worst case scenario.

Age group	Fluoride concentration in toothpaste	Amount of toothpaste used per day	Ingestion rate	Resulting fluoride exposure p.P.
Up to 3 years	1000 ppm	2 g	80%	1.6 mg
6 years	1000 ppm	2 g	36%	0.72 mg
8–10 years	1450 ppm	2 g	30%	0.87 mg
Adults	1500 ppm^a^	2 g	3%	0.09 mg

^a^ Maximum permitted fluoride concentration in toothpaste according to the German Kosmetikverordnung EG 1223/2009.

#### 3.1.2 Detailed Data on Fluoride Ingestion via Mouth Wash

With regard to mouth wash, a twice‐daily usage of 10‐mL mouth wash containing 100 ppm fluoride is assumed for children 6 years and older. For younger children, use of mouth wash is not considered, as it is not recommended for this age group. Adults use 20‐mL mouth wash twice a day, containing 300‐ppm fluoride. Ingestion of mouth wash is calculated based on the ingestion rates given for toothpaste (Barnhart et al. 1974; Cochran et al. 2004; Omid et al. 2017; Zohoori et al. 2012). Considered data are given in Table 2.

**TABLE 2:** Data for mouthwash use considered for the calculation of the daily fluoride intake to represent a realistic worst case scenario.

Age group	Fluoride concentration in mouth wash	Amount of mouthwash used per day	Ingestion rate	Resulting fluoride exposure
Up to 3 years	Not relevant	Not relevant	Not relevant	Not relevant
6 years	100 ppm	20 mL	36%	0.72 mg
8–10 years	100 ppm	20 mL	30%	0.6 mg
Adults	300 ppm	40 mL	3%	0.36 mg

#### 3.1.3 Detailed Data on Fluoride Ingestion via Salt

Daily salt intake is calculated based on data from the BgVV (Bundesinstitut für gesundheitlichen Verbraucherschutz und Veterinärmedizin). Since fluoridated salt is permitted in processed food and convenience food in Germany, a salt consumption of 2 g/day through homemade food is considered (BgVV 2002). The share for children is even smaller. As a realistic worst case, an intake of 1 g is assumed for 6‐ to 10‐year‐olds. The considered data are listed in Table 3.

**TABLE 3:** Data for daily salt intake considered for the calculation of the daily fluoride intake to represent a realistic worst case scenario.

Age group	Fluoride concentration in salt^a^	Consumed salt per day	Resulting total fluoride exposure
Up to 3 years	Not relevant	Not relevant	Not relevant
6 years	310 mg/kg	1 g	0.31 mg
8–10 years	310 mg/kg	1 g	0.31 mg
Adults	310 mg/kg	2 g	0.62 mg

^a^ Maximum permitted concentration for salt in Germany.

#### 3.1.4 Detailed Data on Fluoride Ingestion via Drinking Water

In most European countries, drinking water contains ≤ 0.3 mg/L fluoride. Therefore, a concentration of 0.3 mg/L is assumed as the worst case. The total fluoride intake is calculated based on the reference values for water intake as published by the DGE. Daily water intake considered for the calculation of the daily fluoride intake is given in Table 4.

**TABLE 4:** Data for daily water intake considered for the calculation of the daily fluoride intake to represent a realistic worst case scenario.

Age group	Fluoride concentration in drinking water	Water intake per day	Resulting total fluoride exposure
Up to 3 years	0.3 mg/L	820 mL	0.25 mg
6 years	0.3 mg/L	940 mL	0.28 mg
8–10 years	0.3 mg/L	970 mL	0.29 mg
Adults	0.3 mg/L	1530 mL	0.46 mg

#### 3.1.5 Detailed Data on Fluoride Ingestion via Black Tea and Fish

Foods other than those supplemented with fluoride through salt contain only very small amounts of fluoride. An exception to this is fish and seafood as well as black tea, which can contain fluoride as well. Therefore, both are considered as well.

Cao et al. determined the fluoride content in various black tea commodities and found 1.15–6.01 mg/L in black tea bags (Cao et al. 2006). According to Statista, the average German consumed roughly 28 L of black tea in 2023 (Statista Research Department Forthcoming), which results in a daily consumption of about 0.07 L per day. As a cup of tea contains 200–250 mL, the latter is assumed, resulting in a daily fluoride intake of 1.5 mg from black tea. As children rarely consume black tea due to the caffeine content, black tea consumption is not considered for children up to 10 years.

Fish can contain significant amounts of fluoride as well. Different studies determined the fluoride content in fish and reported concentrations of up to 5 mg/kg (EFSA 2013; Prabhakar and Hegde 2021). According to the supply balance for fish, Germans consumed 13.7 kg fish per year, which results in a daily consumption of 37 g. This is slightly above the German Nutrition Society (DGE) recommendation of 240 g fish per week (DGE 2025), but in good agreement with most national European Food‐Based Dietary Guidelines recommending two servings of about 150 g each per week (EFSA 2014). With a rounded weight of 40 g per day, this would result in an additional fluoride intake of 0.2 mg.

#### 3.1.6 Total Daily Fluoride Intake and Comparison to the EFSA Recommendations

In Table 5, the fluoride intake by source and the resulting calculated total daily fluoride intake—as aggregated exposure—is listed. For comparative reasons, the recommended daily fluoride intake as well as the upper intake level is shown as published by EFSA (EFSA 2006; EFSA 2013). The higher value (calculated vs. recommended intake) for each age group is considered the realistic worst case for the further assessment.

**TABLE 5:** Overview of fluoride intake by source, calculated total intake, recommended intake, and assumed worst case.

Age	Source	Daily fluoride exposure by source (mg)	Total fluoride intake per day (mg)	Recommended fluoride intake per day (mg)	Tolerable upper intake level (mg/day)	Assumed worst case fluoride intake per day (mg)
Up to 3 years	Toothpaste	1.6	**2.05**	0.7	1.5	2.05
	Mouthwash	Not relevant				
	Salt	Not relevant				
	Fish	0.2				
	Black tea	Not relevant				
	Water	0.25				
6 years	Toothpaste	0.72	**2.23**	1.1	2.5	2.23
	Mouthwash	0.72				
	Salt	0.31				
	Fish	0.2				
	Black tea	Not relevant				
	Water	0.28				
8–10 years	Toothpaste	0.87	**2.27**	1.1	5	2.27
	Mouthwash	0.6				
	Salt	0.31				
	Fish	0.2				
	Black tea	Not relevant				
	Water	0.29				
Adults	Toothpaste	0.09	3.23	3.1 (women) **3.8 (men)**	7	3.23 (women) 3.8 (men)
	Mouthwash	0.36				
	Salt	0.62				
	Fish	0.2				
	Black tea	1.5				
	Water	0.46				

*Note:* Bold values represent the assumed worst case intake per day.

Besides the recommended daily intake, the EFSA additionally defined a tolerable upper intake level for fluoride, being 1.5 mg/day for 3‐year‐old children, 2.5 mg/day for 4‐ to 8‐year‐olds, 5 mg/day for 9‐ to 14‐year‐olds, and 7 mg/day for individuals > 15 years (EFSA 2006; EFSA 2013).

For 3‐year‐olds, the calculated worst‐case intake exceeds not only the recommended fluoride intake but also the tolerable upper intake.

### 3.2 Determination of Equivalent Exposure Ranges in Epidemiological Studies

In order to correlate fluoride exposure in Europe with exposure in regions with fluoridated water, the influence of dosage and frequency of exposure needs to be accounted for. Therefore, the pharmacokinetics of fluoride have to be considered.

After oral uptake, fluoride is rapidly absorbed from the gastrointestinal tract and plasma concentrations peak at around 30–60 min after intake. Clearance from plasma takes place through uptake by bone and excretion in urine (NTP 2016) and occurs with a half‐life of 2–9 h (IPCS 2002; Ekstrand 1977; Ekstrand and Ehrnebo 1983). Therefore, severe accumulation does not take place and plasma as well as urinary concentrations are representative for the single dose intake.

As described above, fluoride exposure in Europe is less evenly distributed throughout the day than is the case when exposure occurs through drinking water alone. Both scenarios differ by means of single intake dosages as well as exposure frequency. Therefore, both exposure scenarios cannot be compared based on single‐dose plasma levels.

However, as described by Speirs after exposure of rats (1986), the mean plasma concentration over a given time interval is comparable after continuous fluoride exposure and the same total dose administered as two single doses. Therefore, plasma levels could be considered as a marker for total intake if reported as total daily values. However, since this is generally not the case, but urinary concentrations are more often reported as total daily values in epidemiological studies, these values can be considered as the basis for comparison.

Villa et al. (2010) reviewed existing data to correlate total daily fluoride intake (TDFI) with daily urinary fluoride excretion (DUFE). They concluded that there is a strong relationship between total daily fluoride intake and daily urinary fluoride excretion but with a different slope for children and adults, being 0.35 and 0.54 respectively. The difference between the slopes results from the higher fluoride retention in children's hard tissues compared to adults because of their growing skeletal system (Rugg‐Gunn et al. 2011; NTP 2016).

To calculate the urinary excretion for the estimated daily worst case intake, as given in Table 5, a urinary output of 1–2 mL/kg bw/h for children and 0.5–1 mL/kg bw/h for adults was considered (Nursing Central 2024). With body weights from WHO and CDC growth charts, the urinary output was calculated for 3‐year‐old children and adults (Table 6). The resulting urinary fluoride concentrations for the estimated daily intake are given in Table 7.

**TABLE 6:** Twenty‐four‐hour urine output for 3‐year‐old children and adults.

Age	Urine output (mL/kg bw/h)	Body weight (kg)^a^	Urine output (mL/24 h) (worst case)
3	1–2	14.3	343–686
≥ 19	0.5–1	70.59	847–1694

^a^ Body weights for 3‐ to 10‐year‐old children derived from WHO growth charts (50th percentile, boys), body weights for 13‐ to 20‐year‐old children and adults derived from CDC growth charts (50th percentile, boys/men).

**TABLE 7:** Calculated urine fluoride concentration based on worst case fluoride intake for 3‐year‐old children and adults.

Age	Worst case intake (mg/day)	Excretion rate (%)^a^	Amount of excreted fluoride (mg/day)	Urine volume (mL/day)	Urine fluoride concentration (mg/L/day)
3	2.05	35	0.72	343–686	1.05–2.09
≥ 19	3.8	54	2.05	847–1694	1.21–2.42

^a^ Based on the study of Villa et al. (2010).

Based on the estimated daily fluoride intake in Europe, the daily urinary fluoride excretions are 0.72 mg/day for children and 2.05 mg/day for adults. Considering the urine volume per day results in urinary fluoride concentrations of 1.05–2.09 mg/L/day for children and 1.21–2.42 for adults. The calculated urine fluoride concentrations are in correlation with published data. Haftenberger et al. reported a total daily fluoride intake of 0.48–1.8 mg per day in pre‐school children with a resulting urinary output of 0.42–1.97 mg/L (Haftenberger et al. 2001).

### 3.3 Determination of Equivalent Doses in Animal Studies

In order to correlate fluoride exposure in animals and humans, it needs to be considered that absorption and distribution differ in both species.

For the well‐studied endpoint fluorosis, it could be demonstrated that, despite having differences in metabolism, the adverse effect occurs at similar plasma levels in rats and humans. However, in order to achieve similar plasma levels, rats have to be exposed to much higher doses than humans. According to Angmar‐Månsson and Whitford (1984), disturbances in enamel mineralization in the rat incisor occur at fluoride water concentrations of at least 10 ppm. Similar findings have been described in humans consuming water with fluoride levels of at least 2 ppm (Butler et al. 1985). Dunipace et al. (1995) concluded that rats require about five times greater water concentrations than humans to reach comparable plasma levels.

It is not known but plausible that other effects might also occur at comparable plasma concentrations in both species.

However, as described above, measurements of plasma fluoride concentrations after single‐dose exposure cannot be compared to plasma concentrations during continuous exposure. Therefore, plasma concentrations of rats during exposure through fluoride in drinking water cannot be directly compared to the exposure situation in Europe but only to levels in humans being exposed in the same way, as conducted by Dunipace et al. (1995).

A further stepwise approach is needed to relate animal exposure to the fluoride exposure situation in humans in Europe:

a As it is known that exposure of humans to 2‐ppm fluoride containing drinking water correlates to 10‐ppm fluoride exposure in rats, this concentration is used as a starting point. The daily fluoride intake is calculated for children and adults:

Considering a water intake of 1530 mL/day for adults and 820 mL for 3‐year‐old children as described in Section 3.1.4, the consumption of drinking water with 2 ppm fluoride results in a fluoride intake of 3.06 mg fluoride for adults and 1.64 mg for children.

b For the calculated daily fluoride intake after exposure to drinking water with 2 ppm fluoride, the daily urinary fluoride excretion can be calculated according to the factors given in Villa et al. (2010):

Applying a factor of 0.54 for adults and 0.35 for children results in daily urinary fluoride excretions of 1.65 mg for adults and 0.57 mg for 3‐year‐olds.

c The daily urinary fluoride excretion from people exposed to drinking water with 2‐ppm fluoride can be compared to the daily urinary fluoride excretion from individuals exposed to fluoride from other sources as is typical in Europe (as calculated in Section 3.1.6). The ratio between both exposure situations is determined:

The daily fluoride intake in Europe was calculated to be 3.8 mg for adults and 2.05 mg for 3‐year‐old children. The resulting daily urinary fluoride excretion is 2.05 mg/day for adults and 0.72 mg/day for 3‐year‐old children (Section 3.1.7).

d Comparing the urinary excretion derived through drinking water exposure with 2 ppm fluoride (b) and the European exposure scenario (c) (total daily intake) results in a ratio of 1.25 (rounded value).
e The ratio can be applied to determine an equivalent animal dose, as fluoride intake correlates linearly with both urinary excretion and plasma levels:

The daily fluoride excretion is 1.25 times higher in the European context than after human exposure to drinking water with 2 ppm fluoride, which corresponds to rat exposure at 10 ppm fluoride. Applying this ratio results in an equivalent animal drinking water concentration of 12.5 ppm to model typical European exposure levels.

## 4 Conclusion

In this work, a realistic daily worst‐case fluoride intake was estimated in children and adults based on intake recommendations in Europe. For children, a total daily fluoride intake of 2.05 mg and for adults of 3.8 mg have been estimated. It was found that the calculated worst‐case daily intake in 3‐year‐old children exceeds the current recommendations of fluoride intake by EFSA, and even the tolerable upper intake level solely through toothpaste usage is exceeded, as it is often overdosed and young children have limited disgorging skills. In older children up to 6 years, the recommended daily intake is exceeded when toothpaste, mouthwash, drinking water, salt, and fish consumption are combined. For adults, food also gains more importance as a fluoride source. However, for adults, the calculated realistic daily intake through all sources combined is below the recommended daily intake, so that the latter is assumed as worst‐case.

Despite this, fluoride intake can exceed both the recommended amount and the tolerable upper intake when multiple sources—such as black tea, drinking water with fluoride concentrations above 1 mg/L (used for drinking and cooking), fish, and fluoride tablets—are combined (BfR 2021). Therefore, a combination of all the mentioned sources should be avoided.

After determining the estimated total daily fluoride intake, a correlation was drawn to urinary concentrations in epidemiological studies, considering intake in children up to 3 years as the worst case and intake in adults as the lowest value.

For children, the daily urinary fluoride excretion was calculated to be 0.72 mg/day and for adults 2.05 mg/day. Epidemiological studies reporting comparable urinary levels can be considered to transfer observed effects to the distinct exposure situation in Europe.

However, the calculated values exceed those after exposure to drinking water with 2 ppm fluoride by a factor of 1.25. This is an important finding as exposure to drinking water concentrations above 1.5 mg/L is associated with lower IQ in children (NTP 2024; Health Canada 2023). However, this factor only applies if drinking water is the only fluoride source. In regions with fluoridated water, other sources might also contribute to total fluoride intake. As a result, overall intake could still be higher in regions where lower IQ in children has been observed.

Considering the urine output per day for both age groups gave urinary fluoride concentrations of 1.05–2.09 mg/L/day for children and 1.21–2.42 mg/L/day for adults. However, as the urine volume varies with water intake, exercise, environmental temperature, and nutrient intake, concentrations reported per liter need to be carefully evaluated.

For in vivo studies, rat exposure to fluoride drinking water concentrations of 12.5 ppm was considered to be equivalent to the daily fluoride intake in Europe. However, as plasma concentrations can change with age or length of exposure, an uncertainty factor should be considered. In this study, plasma levels were considered, which are comparable if rats are exposed to drinking water with 5 times higher fluoride concentrations compared to humans. According to the NRC (2006), regarding bone fluoride content after exposure, rats require at least 10‐fold higher fluoride concentrations to achieve comparable levels. Therefore, an additional uncertainty factor of 2 could be proposed, resulting in an equivalent exposure dose of up to 25 ppm in rats.

With the calculated values, the presented approach offers scientists a robust tool to select relevant scientific studies reflecting the fluoride exposure situation in Europe. Reassessing existing studies can deliver important information about possible health effects through chronic exposure to low fluoride levels.

## Conflicts of Interest

The authors declare no conflicts of interest.

## Endnotes

^1^
Fluoride belt: geographical belts with high fluoride contents in groundwater.
